# A Novel Paradigm for Targeting Challenging Targets: Advancing Technologies and Future Directions of Molecular Glue Degraders

**DOI:** 10.3390/molecules31030459

**Published:** 2026-01-28

**Authors:** Yifan Zhang, Linlin Li, Jiajia Xu, Chunchen Che, Jiaqing Jia, Haohao Lu, Qidong You, Xiaoli Xu

**Affiliations:** 1 State Key Laboratory of Natural Medicines, Jiang Su Key Laboratory of Drug Design & Optimization, China Pharmaceutical University, Nanjing 210009, China; spadeking2001@163.com (Y.Z.);; 2 Department of Medicinal Chemistry, School of Pharmacy, China Pharmaceutical University, Nanjing 210009, China

**Keywords:** targeted protein degradation, molecular glue degraders, drug discovery methods

## Abstract

Molecular glue degraders (MGDs) constitute a class of innovative therapeutic agents within the field of targeted protein degradation (TPD). In contrast to proteolysis-targeting chimeras (PROTACs), MGDs induce protein degradation by stabilizing the interaction between an E3 ubiquitin ligase and a target protein. They typically exhibit favorable drug-like characteristics, including lower molecular weight and enhanced bioavailability. Although their discovery was historically serendipitous, recent advances in high-throughput screening, bioinformatics, and artificial intelligence are enabling more systematic identification and optimization. To date, three MGD-based drugs have been approved for clinical use, with numerous candidates under active investigation. This review comprehensively traces the technological progression of MGDs from serendipitous discovery to the current era of rational design. We systematically introduce and critically evaluate strategies for discovering MGDs, accompanied by illustrative examples. Concurrently, we discuss the major challenges hindering the broader application of MGDs and propose potential approaches to address these issues. Finally, we outline prospective research directions in the field. This review aims to provide a holistic framework for understanding the past, present, and future of molecular glue degraders, underscoring their significant potential to reshape the landscape of drug discovery.

## 1. Introduction

The rapid advancements in genomics, proteomics, structural biology, and bioinformatics have facilitated the identification of numerous novel therapeutic targets. Nevertheless, drug development directed against undruggable or difficult-to-drug targets remains a significant challenge [1,2]. These intractable targets are typically characterized by a lack of well-defined binding pockets and insufficient structural information, rendering them refractory to conventional therapeutic approaches [2,3].

In recent years, the field of TPD has garnered substantial interest. TPD has emerged as a promising drug discovery strategy for treating cancers, inflammatory and immune-related diseases, as well as infections [4,5,6]. It has attracted considerable attention due to its broad applicability, potential to target “undruggable” proteins, and capacity to overcome drug resistance [7,8]. Currently, TPD primarily functions via the ubiquitin–proteasome system (UPS) and lysosomal pathways, and can be further categorized into more than ten distinct technological strategies [9,10,11]. These include proteolysis-targeting chimeras (PROTACs) [12], MGDs [13,14], lysosome-targeting chimeras (LYTACs) [15,16], autophagy-targeting chimeras (AUTOTAC) [17], transferrin receptor-targeting chimeras (TransTACs) [18], and chaperone-mediated protein degraders (CHAMPs) [19,20], among others.

PROTACs and MGDs represent two principal modalities for UPS-mediated targeted protein degradation. PROTACs operate by recruiting E3 ubiquitin ligases to facilitate proximity-induced ubiquitination and subsequent degradation of the target protein. In contrast, molecular glues modulate the surface of E3 ubiquitin ligases, promoting or inducing novel protein–protein interactions (PPIs) between the ligase and the target protein, leading to its ubiquitination and degradation. Furthermore, the target specificity and mechanism of action of PROTACs are generally predictable, allowing for rational design based on ligand-target binding models. However, PROTACs often suffer from high molecular weights (MW), poor cell permeability, and unfavorable pharmacokinetic (PK) properties, posing significant challenges for clinical application [14,21,22,23,24].

MGDs enable precise targeting and efficient degradation of the Protein of Interest (POI) [25,26]. Compared to PROTACs, MGDs exhibit a lower molecular weight, enhanced cell membrane permeability, improved oral bioavailability, compliance with the ‘Rule of Five’ for drug-likeness, and generally superior developability profiles. Consequently, MGDs hold immense clinical potential [27,28].

To date, three molecular glue drugs have been approved worldwide: thalidomide and its derivatives (lenalidomide and pomalidomide) [7]. Recognized for their immunomodulatory, anti-inflammatory, and anti-neoplastic effects, these agents have been approved by the U.S. FDA for the treatment of multiple myeloma and other conditions. Additionally, several other MGDs have entered clinical trials. Numerous pharmaceutical companies, including Novartis, Bayer, and Bristol Myers Squibb (BMS), have initiated research programs in the field of molecular glues, underscoring the substantial market potential of these therapeutics [29,30,31] (Table 1).

However, the discovery of MGDs has largely been serendipitous, lacking systematic methodologies and rational design strategies. Current discovery approaches predominantly rely on screening techniques, such as cellular phenotypic screening, PPI-based binding assays, and artificial intelligence-assisted virtual screening [1,25,32,33,34].

In recent years, with the continuous progress in targeted protein degradation technologies, novel molecular glue degraders are being continually developed and translated into clinical applications. This review summarizes the current strategies for MGDs, tracing their evolution from serendipitous discovery towards rational design, and provides a perspective on future directions in this field.

**Table 1:** Information on marketed or clinically investigated MGDs, including chemical structures, research sponsor, development phase, target, and therapeutic indication. Abbreviation: MM: Multiple myeloma. CMML: Chronic myelomonocytic leukemia. R/R AML: Relapsed/Refractory Acute Myeloid Leukemia. HR-LBCL: Untreated high-risk large B-cell lymphoma. RRMM: Relapsed/Refractory Multiple Myeloma. CRC: Colorectal cancer. AML: Acute myeloid leukemia. MDS: Myelodysplastic syndromes. NHL: Non-Hodgkin lymphoma. DLBCL: Diffuse large B-cell lymphoma. * Indicates the proposed structure, which is inferred from patent analysis but has not been officially disclosed.

Pharmaceutical Designation	Chemical Structure	Research Sponsor	Target	Development Phase	Therapeutic Indication	Ref.
Thalidomide	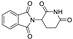	BMS/Celgene	IKZF1/3	launched	MM	[35]
Lenalidomide	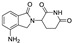	BMS/Celgene	IKZF1/3	launched	MM	[36]
Pomalidomide	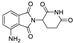	BMS/Celgene	IKZF1/3	launched	MM	[37]
CC-92480	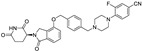	BMS/Celgene	IKZF1/3	Phase III	MM, RRMM	[38]
CC-220	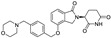	BMS/Celgene	FP91/98;IKZF1	Phase III	MM	[39]
E7820	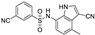	Eisai	RBM39/CD49b	Phase II	CMML, Myeloid neoplasms, R/R AML	[40]
CC-99282	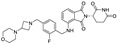	BMS/Celgene	IKZF1/3	Phase III	HR-LBCL	[41]
MRT-2359	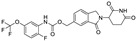	Monte Rosa	GSPT1	Phase I/II	Lung cancer and other solid tumors	[42]
CFT-7455	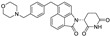	C4 Therapeutics	IKZF1/3	Phase I/II	RRMM	[43]
NVP-DKY709	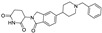	Novartis	IKZF2	Phase I	CRC, Cutaneous melanoma	[44]
CC-90009	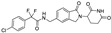	BMS/Celgene	GSPT1	Phase I	AML, MDS	[45]
MRT-6160 *	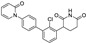	Monte Rosa	VAV1	Phase I/II	Autoimmune disease	[46,47]
SP-3164	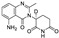	Deuterx LLC	IKZF1/3	Phase I	NHL, DLBCL	[48]
PLX-4545	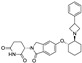	Lexium	IKZF2	Phase I	Multiple tumors	[49]

## 2. Serendipity: From Tragedy to Miracle

Thalidomide, the active ingredient of the drug formerly marketed as Contergan, was initially introduced in the 1950s as a sedative and antiemetic for morning sickness. However, it was withdrawn from the market in 1961 due to its severe teratogenic effects [50,51]. In 1994, the research team of oncologist Judah Folkman discovered that thalidomide inhibits angiogenesis. Folkman proposed a hypothesis that anti-angiogenic agents could suppress tumor growth, and thalidomide served as an excellent molecular candidate to validate this theory [51]. Coincidentally, a patient with multiple myeloma who had not responded to standard therapy was treated with thalidomide at Folkman’s suggestion, resulting in a remarkable therapeutic response. This prompted the initiation of a clinical trial involving 84 patients with relapsed myeloma after chemotherapy. Over one-third of the patients exhibited a response, and 58% survived for more than one year. Encouraged by these results, Celgene conducted larger clinical trials, leading to the accelerated FDA approval in 2006 of thalidomide in combination with chemotherapy as a first-line treatment for multiple myeloma [52].

It was not until 2010 that Japanese scientists identified cereblon (CRBN) as the key target protein of thalidomide [53,54]. Subsequent research gradually elucidated its mechanism of action: by binding to CRBN, thalidomide redirects the CRL4CRBN E3 ubiquitin ligase complex, inducing proteasomal degradation of novel substrates, including the transcription factors Ikaros and Aiolos encoded by the genes IKZF1 and IKZF3. These factors regulate B-cell differentiation and play critical roles in MM, making IMiD-induced degradation of IKZF1/3 essential for the anti-tumor efficacy in MM. In addition, IMiDs exert immunomodulatory effects by enhancing antigen presentation by dendritic cells, increasing production of anti-tumor cytokines, and reducing adhesion molecules on bone marrow stromal cells [55,56].

Lenalidomide, a derivative of thalidomide, exhibits enhanced anti-tumor activity and reduced toxicity. It mediates the degradation of target proteins such as CK1α, demonstrating significant efficacy in hematological malignancies. The development of lenalidomide marks a transition from serendipitous discovery to rational design in molecular glue degraders. Pomalidomide, a third-generation IMiD, offers improved target specificity and a broader therapeutic range. It is not only used in the treatment of multiple myeloma but also employed to modulate CRISPR-Cas9 activity through targeted degradation of Cas9 protein, enabling precise control of gene editing [57,58,59,60].

With growing understanding of the mechanisms of IMiDs, researchers have sought to optimize these molecular glues for enhanced selectivity and potency. Early investigations by Celgene (now acquired by BMS) and BMS into thalidomide analogs have advanced several candidates into clinical development, including **CC-122** [48] for lymphoma, **CC-220** [39] and **CC92480** [38] for relapsed/refractory multiple myeloma, and **CC-90009** [45] for acute myeloid leukemia. Mezigdomide (**CC-99282**) [41], which promotes degradation of IKZF1 and IKZF3 via CRBN E3 ligase engagement, is currently under clinical evaluation both as monotherapy and in combination regimens. **KPG-818** (The chemical structure has not been disclosed yet) [61], a novel orally available CRBN modulator designed by Kling Biopharma with global intellectual property rights, is being investigated in a Phase 1, multicenter, open-label, multiple ascending dose study to evaluate its safety, pharmacokinetics, and preliminary efficacy in combination with dexamethasone in adults with multiple myeloma, as well as a monotherapy in selected hematologic malignancies including mantle cell lymphoma.

**Figure 1:**
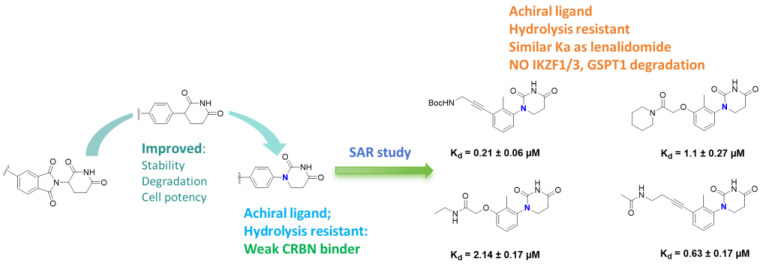
CRBN ligands based on phenyl dihydrouracil derivatives.

Beyond clinically developed CRBN inhibitors, efforts are underway to explore diverse types of CRBN binders. Recently, Tang et al. reported in the *Journal of Medicinal Chemistry* a series of phenyl dihydrouracil derivatives as CRBN ligands. Conventional CRBN ligands, such as thalidomide, lenalidomide, and pomalidomide, are primarily based on glutarimide scaffolds and contain a chiral center prone to racemization. In contrast, phenyl dihydrouracil-based ligands are achiral and avoid stereochemical complications (Figure 1) [62,63].

## 3. High-Throughput Screening (HTS)

High-throughput screening (HTS) plays a critical role in drug discovery, with its multi-level applications encompassing cell-based assays, target-specific screening, and high-content analysis. Through continuous innovation and technological optimization, HTS has not only enhanced the efficiency of drug screening but also provided a powerful platform for research on complex diseases. With further advances in artificial intelligence and big data technologies, HTS is expected to assume an increasingly important role in future drug discovery [64,65,66,67].

In recent years, a number of molecular glue degraders have been identified through HTS-based approaches. These screening strategies can be broadly categorized into two main types: phenotype-based screening and target-based screening [25,33].

### 3.1. High-Throughput Screening Based on Target

Target screening represents a primary and direct strategy for identifying MGDs. This approach detects enhanced binding affinity between the POI and an E3 ubiquitin ligase, facilitating the selection of initial hit compounds. Since both the POI and the E3 ligase are predefined in these assays, target validation is generally not required. However, the requirement for fixed POI–E3 pairs results in a narrower screening window compared to phenotypic methods, often necessitating larger compound libraries to achieve significant results. Common techniques employed in this category include DNA-encoded library (DEL) screening, surface plasmon resonance (SPR), fluorescence resonance energy transfer (FRET) assays, and fluorescent protein complementation assays [2,33,68].

#### 3.1.1. DEL-Based Screening

DNA-encoded library (DEL) technology is a high-throughput screening methodology that involves covalently linking small molecule compounds to unique DNA sequences, thereby creating a compound library tagged with DNA barcodes. These DNA-tagged compounds can be incubated with a target protein, and compounds binding to the target are identified via PCR amplification and sequencing of their associated DNA codes. DELs are combinatorial chemistry-derived small molecule libraries where each compound is uniquely labeled with a DNA tag [69,70]. DELs offer exceptional compound diversity, extremely low synthesis cost per molecule, and provide rich structure-activity relationship (SAR) information. As DEL screening typically relies on affinity-based selection, it is particularly suitable for discovering molecular glues [71].

**Figure 2:**
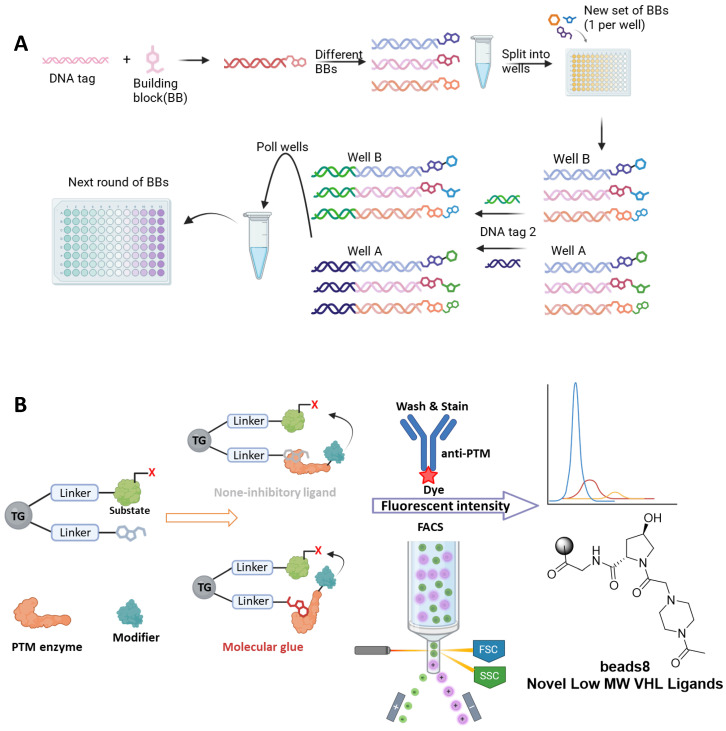
DEL-based screening methods for MGDs: (**A**) construction process of the DEL; (**B**) screening process of the OBOC-DEL.

For molecular glue degrader (MGD) drug discovery, the early-stage R&D team within WuXi AppTec’s Biology Business Unit has developed distinct type of DELs: These libraries are designed based on the core scaffolds of reported small molecule ligands or molecular glues. The design process involves functional group disconnection via retrosynthetic analysis, followed by extensive chemical modifications to significantly expand the chemical space around the core scaffold. These libraries are characterized by their strong target focus, potentially leading to higher success rates. (Figure 2A)

OBOC-DEL is an emerging DEL technology that demonstrates significant advantages over conventional DELs in aspects such as screening efficiency and compound diversity. In OBOC-DEL, each compound is linked to a unique DNA-encoded tag and immobilized on a single bead. This approach facilitates the rapid construction of large-scale, structurally diverse compound libraries using solid-phase synthesis techniques. The synthesis employs a split-and-pool strategy, combining different building blocks to generate all possible compound combinations. The DNA-encoding tags are attached to the beads via enzymatic ligation or click chemistry, ensuring a strict one-to-one correspondence between each compound and its DNA tag [70,71,72].

In August 2025, a team led by Thomas Kodadek at UF Scripps developed an integrated technological platform combining OBOC-based functional screening with an enzyme activity-coupled assay system [73]. This platform enables simultaneous detection of small molecule binding to an enzyme and its functional activity, helping to avoid the selection of mere inhibitors. This is particularly valuable for E3 ubiquitin ligase ligand discovery. Using this platform, Kodadek’s team validated the approach in studies targeting VHL and CRBN, leading to the discovery of a novel, low molecular weight VHL ligand (beads 8, Figure 2B). This technology holds promise for accelerating the development of therapeutic modalities like targeted protein degradation, especially by distinguishing ligand types (simple binders vs. MGs and PROTACs), thereby providing new strategies and tools for drug discovery (Figure 2B).

#### 3.1.2. NanoLuc Binary Technology (NanoBiT)—Based Screening

**Figure 3:**
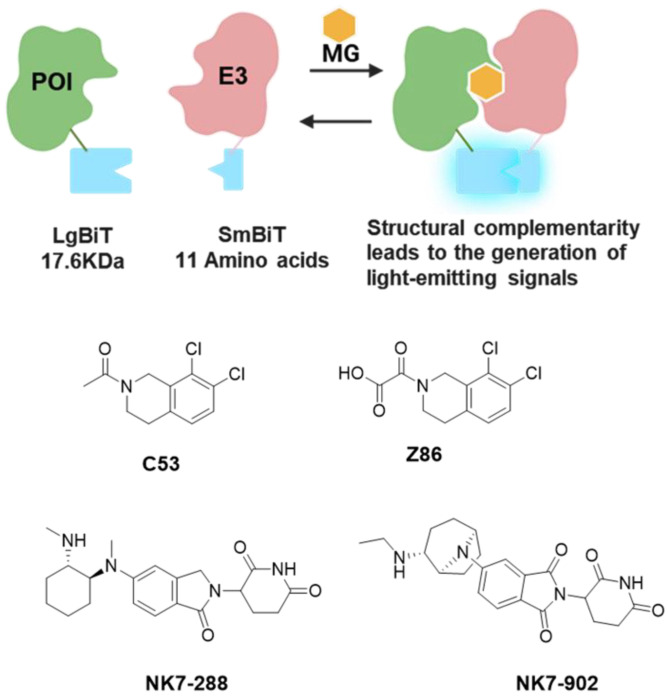
Schematic illustration of the NanoBiT method principle and the structural elucidation of compounds identified through its application in a case study.

NanoBiT enables real-time detection of protein–protein interactions in live cells through a structural complementation approach. In this system, NanoLuc luciferase is split into two optimized subunits: a large subunit (LgBiT, 158 aa) and a small subunit (SmBiT, 11 aa) [74,75]. Both subunits exhibit high stability, minimal self-association affinity, and generate a bright luminescent signal upon complementation. To study protein interactions, LgBiT and SmBiT are genetically fused to a pair of target proteins and co-expressed in cells. When the two target proteins interact, the complementary luciferase subunits reassemble into an active enzyme, producing a bright luminescent signal in the presence of its substrate. The system has been optimized such that the intrinsic affinity between SmBiT and LgBiT is low (Kd∼190 µM), ensuring that complementation is primarily driven by the interaction between the target proteins (Figure 3) [74,76,77].

When applying NanoBiT to a protein pair of interest, it is essential to test all possible fusion orientations to determine the optimal configuration for LgBiT and SmBiT fusions. Plasmids are then transfected into cells using highly efficient, low-toxicity reagents such as ViaFect, FuGENE HD, or FuGENE 6. After an appropriate incubation period, the Nano-Glo^®^ Live Cell Reagent is added, and luminescence is measured using a compatible detection instrument [75].

To identify small molecule enhancers capable of promoting the interaction between LZTR1 and KRAS, thereby facilitating KRAS degradation [78], a team led by Professor Johannes W. Bigenzahn and Professor Giulio Superti-Furga employed the NanoBiT protein–protein interaction assay to screen a library of approximately 450 small molecule fragments. By monitoring the luminescence signal [79], they identified fragment C53 as a significant enhancer of the KRAS-LZTR1 interaction. Subsequent screening of 50 C53 analogs revealed that fragment Z86 also exhibited a moderate enhancing effect. The molecular glue mechanism of these compounds was validated using multiple techniques, including proximity labeling (BioID), thermal shift assays, and NMR spectroscopy, which collectively confirmed their role in stabilizing the LZTR1-KRAS complex. (Figure 3)

In a study reported in July 2025, researchers from Novartis conducted a NanoBiT-based screen of 2545 CRBN-binding molecules. They identified NK7-288 as a compound capable of recruiting NEK7 to CRBN, which also induced weak NEK7 degradation in high-throughput degradation assays [80]. Structural optimization led to the development of NK7-902, which demonstrated specific recruitment of NEK7 to CRBN and potent degradation of NEK7. Structural analysis and key amino acid mutation experiments established that the β-hairpin motif of NEK7 is the critical degradation tag targeted by NK7-902. The compound exhibited robust CRBN/NEK7 recruitment capabilities in both TR-FRET and SPR binding assays. Furthermore, cryo-electron microscopy (cryo-EM) analysis corroborated these findings by confirming the formation of the expected ternary complex. (Figure 3)

#### 3.1.3. Protein Microarrays—Based Screening

Protein microarrays were developed as a functional extension of gene chips. Similarly to DNA microarrays, they consist of high-density arrays of probe proteins or antibodies immobilized on a solid support, which specifically capture target molecules from a sample. Protein microarrays hold significant potential for broad applications and are expected to have substantial impact across various fields including biology, medicine, and environmental monitoring [81,82,83].

**Figure 4:**
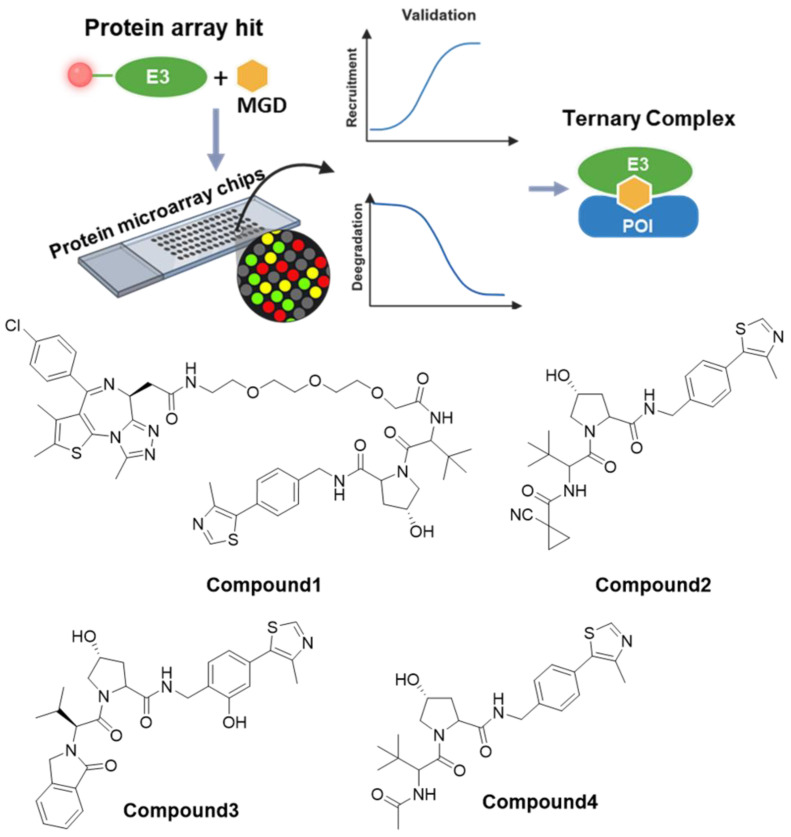
Schematic illustration of the protein microarrays method principle and the structural elucidation of compounds identified through its application in a case study.

Currently, protein microarrays are widely employed in applications such as biomarker detection. Owing to their high-throughput capability, tasks that previously required months or even years—such as biomarker screening—can now be completed within days. Additionally, protein microarray technology is extensively used in studying biomolecular interactions, investigating drug targets and mechanisms of action, and exploring protein functions (Figure 4) [81,84].

In April 2024, Novartis employed this technology to discover a VHL-based molecular glue targeting CDO1 [85]. Although both CRBN and VHL ligands are among the most commonly used E3 ligase recruiters, no highly synergistic small-molecule glue degraders targeting VHL had been reported prior to this study. The researchers first used protein microarrays to identify a PROTAC molecule capable of inducing proximity between VHL and BRD4. After validating the approach, they tested a mixture of Compounds **1**, **2**, **3**, and **4**. (Figure 4) Results indicated that, compared to the DMSO control, the mixture-treated group showed a strong signal for CDO1. To identify which compound was responsible, they expressed and purified full-length CDO1 and performed surface plasmon resonance (SPR) assays. The results demonstrated that Compound **4** (Figure 4) reached binding saturation at 10 µM (KD = 0.82 µM). Further confirmation via TR-FRET and 2D-NMR experiments indicated that Compound **4** acts as a molecular glue by facilitating the formation of a ternary complex between VHL and CDO1.

#### 3.1.4. FRET&BRET-Based Screening

**Figure 5:**
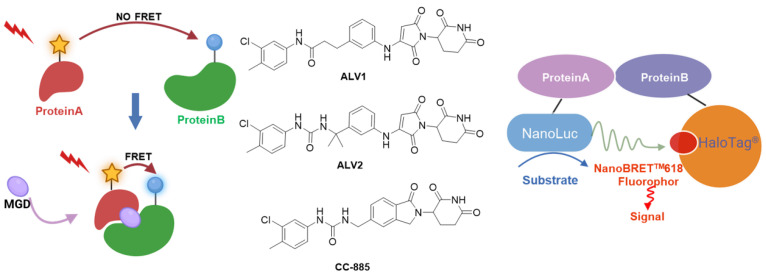
Schematic illustration of the FRET&BRET method principle and the structural elucidation of compounds identified through its application in a case study.

Fluorescence resonance energy transfer (FRET) is a non-radiative, fluorescence-based technique widely employed to investigate molecular interactions and distance changes at the nanometer scale. FRET relies on energy transfer between two fluorophores: a donor and an acceptor. When the donor molecule is excited, it can non-radiatively transfer energy to an acceptor molecule if the latter is within close proximity (typically 1–10 nm), resulting in a decrease in donor fluorescence and an increase in acceptor emission. FRET can be performed in live cells without the need for physical labeling, offering high sensitivity and specificity [86,87] (Figure 5).

Bioluminescence resonance energy transfer (BRET) operates on a similar principle but utilizes a bioluminescent protein (e.g., NanoLuc luciferase) as the donor instead of a fluorophore. Compared to FRET, BRET offers several advantages: it does not require external excitation light, thereby minimizing background noise and autofluorescence; it reduces photobleaching and causes less cellular damage; and the small size of NanoLuc facilitates its use in fusion protein constructs while providing strong luminescent output. This method is commonly applied to detect compound-induced PPIs [88,89] (Figure 5).

Wang et al. established a FRET-based screening platform to monitor IKZF2–CRBN dimerization and, through structure-guided optimization supported by protein crystallography, successfully developed the selective IKZF2 molecular glues ALV1 and ALV2 (Figure 5) [90]. Compared to lenalidomide and CC-885 (Figure 5), both compounds induce significantly stronger CRBN–Helios interactions.

### 3.2. High Throughput Screening Based on Phenotype

Phenotypic drug screening is a drug discovery approach based on observable changes in the phenotype of biological systems, and it has proven to be a highly effective and powerful strategy for identifying molecular glue degraders. This method does not rely on prior knowledge of specific target proteins or their druggability. Instead, it identifies active molecules by observing the effects of compounds on the overall phenotype of cells or organisms—such as cell viability, expression of specific proteins, or alterations in disease-relevant pathways. It is particularly well-suited for discovering degraders with novel mechanisms of action [91,92,93].

In phenotypic drug screening, active compounds engage biological systems through often unknown mechanisms, activating physiologically relevant pathways or cellular signaling events. This approach facilitates the discovery of novel signaling cascades, molecular mechanisms, and therapeutic targets, thereby expanding the repertoire of druggable targets and regulatory mechanisms. However, a major limitation of phenotypic screening is that the specific targets and mechanisms of action of hit compounds frequently remain unidentified, which complicates subsequent drug development efforts [94,95,96]. Deconvoluting the molecular targets of active compounds is a highly challenging process, and even when a putative target is identified, further validation remains essential.

#### 3.2.1. Cell Line-Based Toxicity Screening

**Figure 6:**
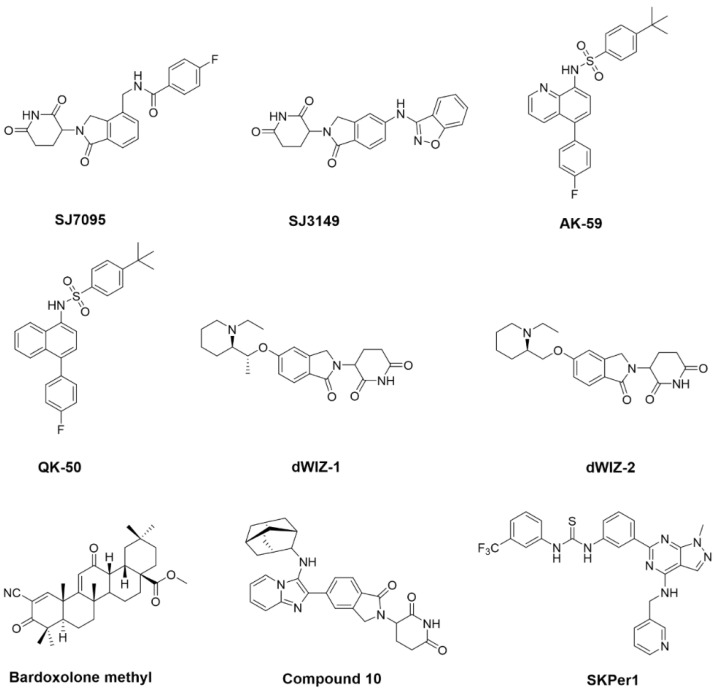
Structures of compounds identified through phenotypic screening for MGDs.

In January 2024, a research team led by Professors Marcus Fischer, Jeffery M. Klco, and Zoran Rankovic at St. Jude Children’s Research Hospital (USA) conducted a screening of a 3630-compound molecular glue library across nine pediatric cancer cell lines (MB002, MB004, HD-MB03, MHHCALL-4, MOLM-13, TF-1, HEL, OCI-AML3, and AML193). By clustering the activity profiles across these cell lines, they identified compound SJ7095 (Figure 6) as exhibiting potent cytotoxicity and high selectivity toward MOLM-13 cells [97].

Using TMT-based quantitative proteomics, the team identified CK1α, IKZF1, and IKZF3 as substrates degraded upon SJ7095 treatment. Subsequent molecular dynamics simulations and structure-activity relationship (SAR) studies enabled the development of SJ3149 (Figure 6)—a compound that selectively degrades CK1α both in vitro and in vivo. SJ3149 demonstrated broad-spectrum anti-proliferative activity against both hematological malignancies and solid tumors.

#### 3.2.2. Signal Pathway-Based Regulatory Screening

In May 2024, a team led by Danilo Guerin at the Novartis Institute for Biomedical Research (Switzerland) identified AK59 (Figure 6), a small-molecule degrader of STING. Initially, the team screened and identified AK59 as a potential STING inhibitor, along with its structural analog QK50 (Figure 6) [98]. IRF reporter assays, Western blot analysis and Mass spectrometry results indicated that AK59 promotes STING degradation via the ubiquitinproteasome system (UPS), thereby inhibiting the cGAS/STING signaling pathway.

To identify genes involved in AK59-induced STING degradation, the team performed a genome-wide CRISPR knockout screen and quantitative proteomic profiling. Significant enrichment was observed for the E3 ligase HERC4, as well as the ubiquitin-like modifier activating enzymes UBA5 and UBA6. A NanoBiT complementation assay confirmed that AK59 induced a time- and dose-dependent increase in luminescence, indicating enhanced interaction between STING and HERC4 and supporting a molecular glue mechanism that facilitates ternary complex formation.

In July of the same year, the same research group reported the discovery of molecular glue degraders dWIZ-1 and dWIZ-2, which target the transcription factor WIZ—a previously unrecognized suppressor of fetal hemoglobin (HbF) [99]. These compounds show promise as potential therapeutics for sickle cell disease (SCD). To identify small molecules that induce HbF expression, the researchers first conducted a high-throughput screen of a library containing 2841 CRBN-biased molecules. They identified compound C (dWIZ-1, Figure 6), which elevated HbF levels via a degradation-dependent mechanism. An unbiased proteomic stability assay using mass spectrometry revealed that, among 8960 quantified proteins, WIZ was the most significantly downregulated target. Further studies confirmed that dWIZ-1 acts selectively on WIZ.

Using AlphaFold-based sequence analysis and structural modeling, the team identified putative zinc finger (ZF) domains in WIZ. Experimental validation supported a mechanism in which dWIZ-1 recruits WIZ (via ZF7) to the CRBN-DDB1 E3 ligase complex, triggering targeted protein degradation in a dose-dependent manner. To extend these findings in vivo, the team developed an optimized analog, dWIZ-2 (Figure 6), in which the chiral methyl group of dWIZ-1 was removed to improve pharmacokinetic (PK) properties. dWIZ-2 effectively degraded WIZ in primary human erythroblasts in vitro. Moreover, dose-dependent WIZ degradation was observed in erythroblasts derived from CD34+ cells of three SCD patients. Treatment with dWIZ-2 increased both the percentage of HbF-expressing cells and total HbF levels in SCD-derived erythroid cells, supporting its therapeutic potential.

#### 3.2.3. Natural Products

Bardoxolone methyl (CDDO-Me, Figure 6) is a synthetic triterpenoid derived from the natural product oleanolic acid. It functions as an inhibitor of IKK, suppressing NF-κB transcriptional activity, and also acts as an activator of the Keap1–Nrf2 pathway, contributing to its anti-inflammatory and anti-cancer properties. Clinically, it has been investigated for the treatment of solid tumors, type II diabetes, and chronic kidney disease [100].

In December 2024, a research team led by Bao at the Kunming Institute of Zoology, Chinese Academy of Sciences, identified bardoxolone methyl as a molecular glue degrader [101]. The team established a phenotype-based high-throughput screening platform to evaluate 1956 compounds in HEK293T cells overexpressing EGFR-GFP. The primary readouts included reduction in GFP fluorescence intensity and effects on cell viability in MDA-MB-231 cells. Through this approach, 89 compounds were identified that significantly decreased GFP fluorescence. Subsequent cell viability assays confirmed CDDO-Me as the most potent compound in reducing EGFR protein levels.

Mechanistic investigations revealed that CDDO-Me directly interacts with the tyrosine kinase (TK) domain of EGFR, promoting its association with KEAP1—an adaptor for the Cullin3-based E3 ubiquitin ligase complex. This interaction facilitates ubiquitination of EGFR, particularly via K63-linked polyubiquitination, which marks the receptor for degradation through the autophagy-lysosome pathway.

#### 3.2.4. Multi-Component-Based Screening

Multicomponent orthogonal synthesis is an efficient and modular chemical strategy designed to rapidly construct diverse compound libraries by simultaneously introducing multiple reactive components within a single reaction system. The core concept of this approach lies in leveraging orthogonal reactions—where multiple chemical transformations proceed concurrently without interference under identical conditions—enabling efficient combination and conversion of multiple building blocks [102,103,104]. In orthogonal synthesis, each component typically serves a specific function, such as acting as a core scaffold, a linker unit, or a functional group. Through precise design of reaction conditions and component combinations, large libraries of structurally diverse and functionally well-defined compounds can be generated in a short time. Importantly, libraries synthesized via this method often require minimal purification and can be directly used in subsequent biological assays or screening campaigns [105,106].

This methodology finds broad applications in drug discovery, materials science, and chemical biology, offering particular advantages in high-throughput screening and structure-activity relationship (SAR) studies.

In November 2024, a collaborative team led by Prof. Nathanael S. Gray from Stanford University and Prof. Eric S. Fischer from Harvard Medical School introduced a strategy based on multicomponent reactions to systematically explore chemical space, enabling efficient screening and identification of potential target proteins such as WEE1 and CK1α. Using the Groebke–Blackburn–Bienaymé (GBB) multicomponent reaction, they synthesized a library of compounds containing glutarimide motifs. The GBB reaction is a highly efficient one-pot process that utilizes simple precursors—such as an aldehyde, an aminoazine, and an isocyanide—to generate structurally diverse products in a single step [107].

Through this approach, the team synthesized over 200 compounds. Subsequent cell proliferation assays in the acute lymphoblastic leukemia cell line MOLT-4 identified a candidate molecule, compound 10 (HRZ-1, Figure 6), which exhibited significant anti-proliferative activity. The molecular glue mechanism of HRZ-1 was further validated using TR-FRET assays and cryo-EM.

#### 3.2.5. Cell Viability to Death Phenotypic-Based Screening—The DEFUSE Platform

In September 2025, a collaborative effort between the research teams of Jiang Hai and Deng Xianming led to the development of a novel high-throughput screening platform for protein degraders, named DEFUSE (Death Fusion Escaper). The researchers fused the target protein to a rapidly activatable death protein, FKBP12 F36V–CASP9, and transduced the genetic construct encoding this fusion protein into cells to generate stable expression cell lines [108]. Upon activation of the death module, cells undergo rapid death within hours. However, if a compound capable of inducing targeted protein degradation is present in the screening library, the death protein is co-degraded, thereby promoting cell survival. This approach converts target protein degradation into a visually detectable viability-to-death phenotypic switch, indicating the presence of a degrader molecule.

Using this high-throughput screening approach, approximately 8000 compounds were screened, the team identified a small molecule, termed SKPer1 (Figure 6), which induces the degradation of the oncoprotein SKP2 and selectively kills SKP2-expressing cancer cells. Mechanistically, SKPer1 functions as a novel MGD that does not directly inhibit SKP2, but instead recruits it to the ubiquitin ligase STUB1, facilitating their interaction and leading to SKP2 ubiquitination and subsequent degradation. SKPer1 demonstrated significant tumor-suppressive effects in vivo with a favorable safety profile. Furthermore, the team showed that a 10-amino acid peptide sequence derived from SKP2 can serve as a universal degradation tag. When fused to other proteins of interest, this tag enables their recruitment to STUB1 and degradation via SKPer1.

### 3.3. Comparison and Selection of Strategies for Phenotypic Screening and Target-Based Screening

The discovery of molecular glue degraders presents both challenges and opportunities. Phenotypic screening and target-based high-throughput screening represent two distinct high-throughput screening (HTS) methodologies, each with its unique application scenarios, advantages, and limitations. Understanding when to prioritize one approach over the other, as well as their differences in terms of false positive risks, scalability, and ease of use, is crucial for advancing drug discovery efficiently [109].

#### 3.3.1. Comparison of Applicable Scenarios

Phenotypic screening follows a “biological-first” principle, which does not presuppose specific protein targets but identifies active molecules by observing the effects of compounds on cellular phenotypes, such as cell viability, specific protein expression, and alterations in signaling pathways. In the discovery of molecular glue degraders, phenotypic screening should be prioritized in the following situations:

**Lack of Target Information or “Undruggable” Targets**: When the target protein lacks a clear ligand binding pocket or when there is limited understanding of its structure and function, rational design based on target information becomes challenging. Phenotypic screening can overcome this barrier by identifying breakthroughs directly at the cellular functional level. For instance, the translation termination factor GSPT1 was initially considered “undruggable,” but through phenotypic screening, Cereblon E3 ligase modulators were identified, successfully achieving its degradation and providing new strategies for the treatment of related cancers [110]. **Aiming to Discover Novel Biology or New Targets**: The greatest advantage of phenotypic screening lies in its “ready-to-use” potential, which can unveil entirely new protein degradation relationships and biological mechanisms. For example, through phenotypic screening of a library of CRBN-biased compounds, researchers unexpectedly discovered that the transcription factor WIZ serves as a previously unknown repressor of fetal hemoglobin, opening new avenues for sickle cell disease treatment [99]. This approach does not rely on existing knowledge and can expand the boundaries of the “degradable proteome.” **Need for Novel Chemical and Biological Starting Points Early in the Project**: When traditional methods based on known target-ligand interactions encounter bottlenecks, phenotypic screening can provide novel chemical structures and mechanisms of action as new starting points, enhancing optimization possibilities [111].

In contrast, target-based HTS adopts a “target-first” strategy, requiring prior identification of the target protein and the establishment of biochemical or biophysical assay methods for that target (e.g., enzyme activity, protein–protein interactions). This method is more suitable under the following circumstances:

**Clear Targets with Established Assays**: When disease-related target proteins are well-validated, and their activity or interactions can be stably and reliably assessed in vitro, target-based HTS is direct and efficient. This approach allows for the rapid screening of large compound libraries to identify hits that bind directly to the target. **Aiming to Optimize Known Degraders or Explore the Potential of Known E3 Ligases**: If a hit degrader has already been identified through other means (such as serendipitous discovery or phenotypic screening), but its potency and selectivity need enhancement, rational design and target-based screening can be optimized based on the known ternary complex structure formed between the degrader and the target protein/E3 ligase. For instance, optimizing hit compounds through X-ray crystallography and computational modeling can lead to significant improvements in potency, selectivity, and in vivo exposure. **Need for Highly Specific Degraders**: Target-based screening, especially when guided by structural information, is more likely to design degraders with high specificity for a given target, thereby reducing the risk of off-target effects.

This comprehensive understanding aids researchers in making informed decisions about which screening approach to utilize, fostering more efficient pathways in drug discovery.

#### 3.3.2. Comparison of False Positive Risks and Challenges

The false positive risks associated with phenotypic screening are primarily twofold: First, the relationship between phenotypic activity and degradation may not be direct. The foremost challenge lies in distinguishing whether the observed phenotypic changes are genuinely driven by the degradation of the target protein. Compounds may produce similar phenotypes through off-target mechanisms, such as inhibition, activation, or other toxic effects. Second, the complexity of mechanism elucidation presents significant difficulties. Even if degradation events are confirmed, subsequent identification of the specific degraded target proteins and clarification of how molecular glues mediate the interaction between E3 ligases and target proteins are highly intricate and time-consuming processes. This necessitates the use of various techniques, including chemical proteomics and CRISPR screening, for thorough investigation [109].

Similarly, target-based screening also carries two main false positive risks: First, binding activity does not equate to degradation. Compounds that can bind to the target at a biochemical level may not necessarily form effective ternary complexes in a cellular environment that lead to degradation. They may lack the ability to recruit E3 ligases, or their binding modes may hinder ubiquitin transfer. Second, binding activity validated in vitro may not translate to effective degradation due to poor cellular permeability, metabolic instability of the compound, or occurrence of other interactions within the cell, potentially leading to unexpected toxicity [112].

#### 3.3.3. Analysis of Scalability and Usability

The scalability of phenotypic screening has significantly improved due to advancements in technological platforms. Integrated platforms, such as workflows that combine solid-phase parallel synthesis with direct biological screening, can rapidly generate libraries of bifunctional molecules with rich chemical diversity for efficient screening. Automated, miniaturized synthesis platforms (e.g., Rapid-Glue) enable the rapid construction of thousands of molecular glue libraries for direct cellular screening without the need for purification, greatly enhancing the efficiency of discovering new molecular glues [93]. Machine learning-assisted analyses also facilitate the handling of high-throughput phenotypic data. Thus, the scalability of modern phenotypic screening is quite robust. For researchers, directly conducting cellular experiments to observe phenotypic changes is intuitive and relatively easy to implement. However, the complete phenotypic screening discovery process (library construction, screening, and target elucidation) demands high interdisciplinary collaboration (encompassing chemistry, biology, and proteomics) and is generally more complex.

In contrast, target-based screening is highly scalable and automated once the target and detection methods are established. It allows for the screening of ultra-large compound libraries at a very low cost. However, the challenge for molecular glue research lies in the initial development of biochemical assays capable of simulating ternary complex formation or evaluating the functionality of “molecular glues,” which is inherently difficult. Once a stable detection system is established, its operation is relatively standardized and straightforward. Nonetheless, it is heavily dependent on prior target validation and assay development; if the target is unsuitable for assay establishment, the method cannot be initiated. Furthermore, it requires the prior acquisition of target proteins or the clever design of detection principles.

In the future, as synthetic platforms, detection technologies, omics analyses, and computational tools continue to advance, the boundaries between these two screening strategies will gradually blur. Intelligent, data-driven discovery platforms that integrate the strengths of both approaches are likely to become the mainstream in the development of molecular glue degraders.

## 4. Informatics Method Analysis and Screening

Drug discovery is a critical phase in the pharmaceutical R&D pipeline. Bioinformatics technologies facilitate the identification of candidate drug molecules by analyzing large-scale biological data, such as genomic, proteomic, and metabolomic datasets. These approaches enable researchers to elucidate disease mechanisms at the molecular level, identify potential drug targets, and predict the binding affinity of small molecules to these targets [113,114,115]. For instance, genomic data analysis allows for the identification of genetic variants associated with specific diseases, thereby supporting the screening of potentially therapeutic compounds [116,117,118].

**Figure 7:**
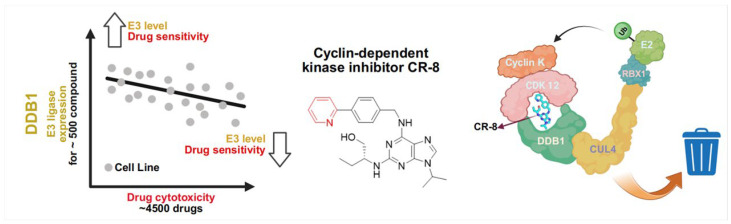
Discovery of CR-8 using bioinformatics and computational approaches.

In 2020, Ebert and Thoma et al. employed bioinformatic methods to investigate 4518 drugs and clinical molecules, analyzing their sensitivity correlations across 578 cancer cell lines and the mRNA expression levels of 499 E3 ligases [119]. Their integrated computational and experimental approach identified CR8 (Figure 7) as a potential molecular glue degrader. Subsequent functional genomics analyses revealed that CR8-induced degradation of cyclin K requires components of the DDB1 complex—including CUL4B, NEDD8, UBE2A, and RBX1—yet notably, no canonical E3 ligase substrate receptor was identified. Instead, CDK12, a kinase unrelated to E3 ligases, was found to be essential for cyclin K degradation and acts as a CR8-binding partner.

Pull-down and TR-FRET assays demonstrated that although CDK12 exhibits very weak intrinsic affinity for DDB1, the presence of CR8 enhances this interaction by 500–1000-fold. Structural elucidation of the DDB1–CR8–CDK12 complex at 3.5 Å resolution revealed an extensive protein-protein interface spanning 2100 Å^2^, stabilized by CR8 acting as a molecular bridge. Structural analysis indicated that CR8 engages the beta-propeller domain of DDB1 via its hydrophobic phenylpyridyl ring system. This interaction redirects cyclin K to the ubiquitination zone typically occupied by degradation substrates, thereby demonstrating that CR8 functions as a molecular glue that bypasses the need for a dedicated E3 substrate receptor and hijacks the DDB1 adaptor to induce target degradation (Figure 7).

Following their seminal 2020 study on CR8, the teams led by Benjamin L. Ebert and Nicolas H. Thomä published a paper titled “Design principles for cyclin K molecular glue degraders”. This work delineates the structural and interaction mechanisms by which CDK inhibitors promote cyclin K degradation. By comparing crystal structures of diverse cyclin K degraders with varying chemotypes, the authors established a structural framework for rational design of molecular glues targeting cyclin K, providing valuable insights for future drug development efforts [120,121,122,123].

## 5. AI-Based Prediction

In recent years, artificial intelligence (AI) technologies have been increasingly applied in drug discovery, demonstrating significant potential in the identification and optimization of molecular glue degraders. The application of AI in molecular glue degrader development primarily focuses on three aspects: prediction of molecular glues, virtual screening and design, and mechanistic investigation and optimization [124,125,126,127].

**Figure 8:**
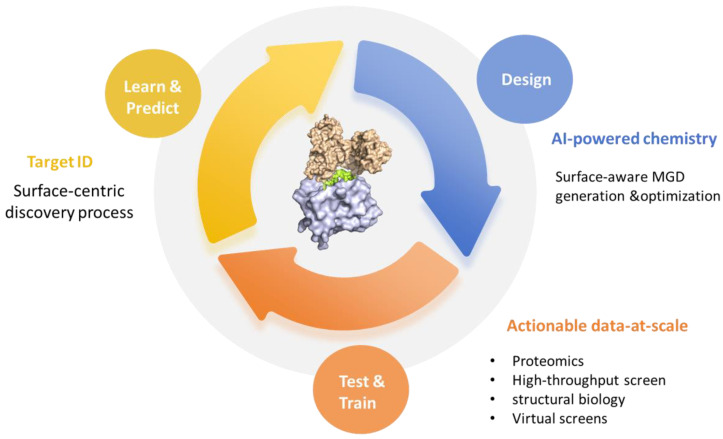
QuEEN™ platform for molecular glue degrader discovery.

Established in 2019, Monte Rosa is one of the pioneering companies in developing molecular glue degraders. Its drug discovery engine, QuEEN™ (Quantitative and Engineered Elimination of Neosubstrates), integrates AI-guided chemistry, diverse chemical libraries, structural biology, and proteomics to enable the rational design of molecular glue degraders. The QuEEN platform first established a proprietary chemical library containing over 10,000 structures specifically designed for reprogramming ubiquitin ligases. These compounds are engineered to interact with E3 ubiquitin ligases such as CRBN, thereby inducing targeted protein degradation. By incorporating AI technology, the QuEEN platform has developed predictive models to assess the activity and selectivity of molecular glue degraders, which accelerates the drug discovery process and reduces experimental costs and time. The QuEEN platform consists of three main modules: (1) a CRBN ligand-derived molecular library, (2) proximity screening technology coupled with chemoproteomic validation, and (3) AI-based predictive models (Figure 8).

The driving mechanisms of CRBN new substrate specificity have become a research hotspot in the field of targeted protein degradation in recent years. The core function of certain MGDs is to induce CRBN to generate a new molecular surface, thereby recruiting and ubiquitinating proteins that are not its natural substrates, referred to as “new substrates.” Early studies established that new substrates bind to the CRBN-drug complex through a conserved structural feature. For example, GSPT1 interacts with CRBN and CC-885 via a key glycine residue located in its surface turn. More systematic studies have found that known CRBN new substrates share a generalizable β-hairpin G-loop recognition motif. Based on this motif, computational mining has predicted over 1600 potential CRBN-compatible proteins in the human proteome and identified a new helical G-loop motif, significantly broadening the potential target space for CRBN [128].

**Figure 9:**
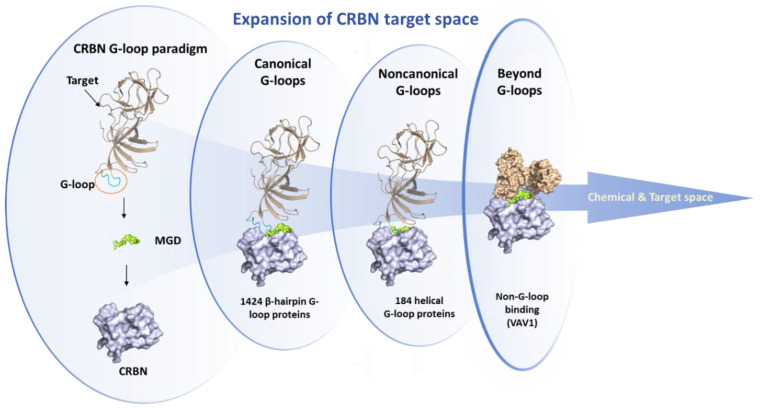
Expansion of the CRBN-mediated targetable degradable proteome through computational discovery of diverse recognition motifs.

However, recent research has challenged this classical paradigm, revealing more diverse recognition mechanisms: certain new substrates do not directly adopt the classic G-loop motif. For instance, VAV1 binds to CRBN through a molecular surface mimicry mechanism (Figure 9) [128]. More typically, G3BP2 bypasses the classical interaction with the CRBN CULT domain, instead binding to an unconventional site on the LON domain of CRBN. The interface of its ternary complex does not resemble known CRBN interactions; rather, CRBN mimics the “footprint” of G3BP2′s endogenous binding partner, utilizing pre-existing protein–protein interaction hotspots on the target protein [129]. This type of mimicry and stabilization of the natural PPI “footprint” is referred to as “glueprints,” providing new strategies for rationally expanding the MGD target library.

The binding of new substrates mediated by IMiDs or CELMoDs closely mimics the recognition pattern of natural CRBN degradation determinants. In crystal structures, the natural degradation determinant peptide forms a fine network of hydrogen bonds with CRBN through the backbone of its C-terminal six residues, without involving side chains. New substrates achieve binding by mimicking each hydrogen bond in this network, explaining why the recognition of new substrates is largely independent of amino acid sequence [130].

CRBN is not a static “hook”; the dynamic conformational changes it undergoes are crucial for the recruitment of new substrates. Cryo-electron microscopy studies have shown that the binding of CELMoD compounds to the thalidomide binding domain (TBD) of CRBN is both necessary and sufficient to trigger CRBN’s conformational rearrangement from an open conformation (CRBN open) to a classic closed conformation (CRBN closed). The new substrate Ikaros can only stably bind to the closed conformation of CRBN, underscoring the importance of conformational effects on the efficacy of CELMoD compounds [131]. In addition to the orthosteric binding site, there exists an evolutionarily conserved hidden allosteric binding site on CRBN [132]. Upon binding of the small molecule SB-405483 to this site, it can synergistically enhance the binding of orthosteric ligands and alter their new substrate degradation profiles. Structural studies indicate that this allosteric ligand functions by shifting the distribution of the open conformation of CRBN towards a new CRBN int conformation and increasing the closed status of CRBN. This finding opens new avenues for enhancing the selectivity and efficacy of CRBN therapies through allosteric modulation.

Current research on the driving mechanisms of CRBN new substrate specificity has entered a multifaceted and dynamic in-depth phase. The understanding of mechanisms has expanded from initially shared motifs (such as G-loop) to a comprehensive model that includes molecular surface mimicry, allosteric regulation, and the analysis of complex ternary complex interfaces [128,132]. The exploration of ligand chemical space, combined with high-throughput screening technologies, is rapidly expanding the known new substrate landscape at an unprecedented pace. These advances not only explain the efficacy and toxicity of existing drugs but also provide new insights into overcoming clinical resistance.

On 10 October 2025, the team led by Lai and Pei from the Center for Quantitative Biology at the Academy for Advanced Interdisciplinary Studies, Peking University, systematically compiled and organized MG-PDB, the most comprehensive dataset of molecular glue ternary complex structures to date. From this, they constructed MGBench, the first benchmark designed to evaluate the generalization capability of structure prediction models. Through a comprehensive and rigorous benchmark evaluation of five mainstream AI co-folding models, including AlphaFold 3, the study delineated the current capabilities and core challenges of existing methods in predicting molecular glue structures. This work lays a foundation for the future development of next-generation computational methods for molecular glue design [133,134].

While AI has demonstrated remarkable capabilities in predicting ternary complex structures, in-depth sequence and structural homology analyses suggest that many of its successful predictions stem from “memorizing” interaction patterns present in the training data rather than genuinely learning atomic-level interaction principles. Consequently, when confronted with entirely novel E3 ligase systems or interaction motifs absent from the training dataset, the predictive accuracy of these models declines significantly [135].

Furthermore, experimental structural data for molecular glue-induced ternary complexes remain scarce. Although benchmark datasets such as MG-PDB have been established, their scale is still insufficient for training robust AI models. Future AI models must move beyond existing general-purpose protein co-folding frameworks and be specifically trained and optimized for molecular glue-mediated protein–protein interactions. Emphasis should be placed on enhancing the model’s ability to generalize and reason about novel interaction patterns, rather than merely improving its recall of known ones.

Simultaneously, AI models should be more deeply and multi-dimensionally integrated with transcriptomic, proteomic, and structural biology data to generate more reliable predictions [136]. Additionally, the research scope should extend beyond conventional proteasome-mediated degradation pathways. For example, exploring the design of molecular glues that target lysosomal degradation could offer new strategies for degrading membrane proteins and extracellular proteins [137].

## 6. Degron-Targeting Strategy

**Figure 10:**
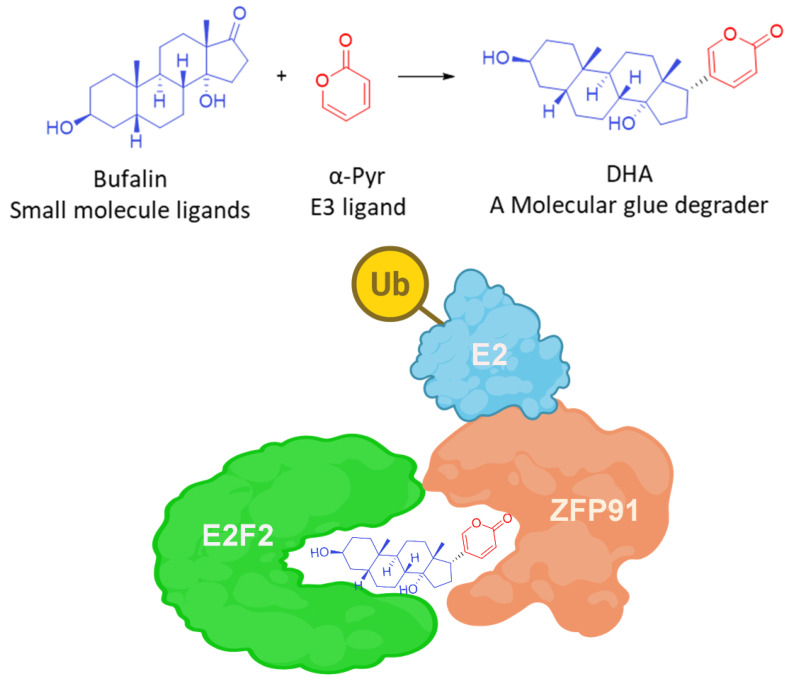
Degron-targeting strategy—using bufalin as an example.

The Degron-Targeting design strategy involves modifying a small-molecule inhibitor (or a target protein-binding ligand) by introducing a minimal E3 ligase-recruiting fragment [32]—such as the glutarimide moiety for CRBN or the α-pyrrole fragment for ZFP91 (as seen in natural products like bufalin)—at specific positions, thereby conferring molecular glue functionality [138,139] (Figure 10).

**Figure 11:**
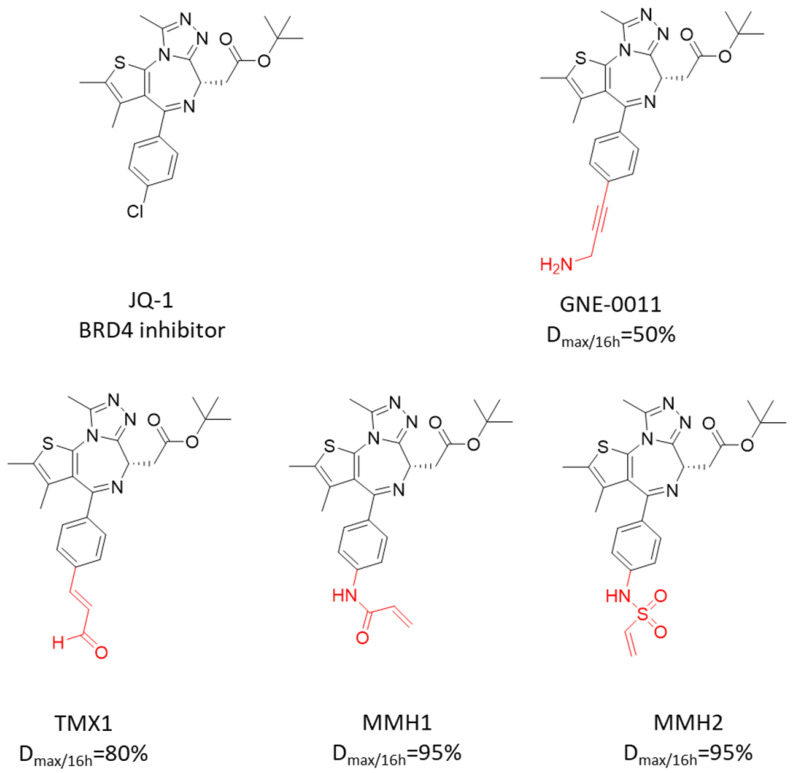
Genentech’s research.

At the 2019 AACR conference, Genentech presented an abstract titled “GNE-0011, a novel monovalent BRD4 degrader,” reporting a monovalent BRD4 degrader developed using this “degrader tail” strategy. GNE-0011 selectively degraded BRD4 through a mechanism dependent on recruitment of the E3 ligase DCAF16, leading to suppression of downstream MYC expression. In vivo, GNE-0011 administered at 10 mpk induced significant tumor regression in an EOL-1 eosinophilic leukemia xenograft model, outperforming JQ1. However, it also exhibited notable toxicity even at doses lower than those of JQ1. Structurally, GNE-0011 replaces the terminal chlorine on the phenyl ring of JQ1 with an amino propynyl group, enabling covalent recruitment of DCAF16 and subsequent degradation of BRD4. Compared to the BRD4-DCAF16 PROTAC molecule KB0-JQ1, GNE-0011 has a substantially reduced molecular weight and shows over 1000-fold improvement in potency. (Figure 11)

In February 2023, a preprint entitled “Template-assisted covalent modification of DCAF16 underlies activity of BRD4 molecular glue degraders” was published on BioRxiv. This study further investigated the structure-activity relationship (SAR) and mechanism of GNE-0011, introducing a “template-assisted covalent modification” strategy. The authors confirmed that GNE-0011 specifically induces degradation of proteins containing the second bromodomain of BRD4, achieving a maximum degradation depth of approximately 50% after 16 h [140]. Through systematic modification of the electrophilic warhead, they elucidated the mechanism and identified novel chemical handles for the rational design of molecular glue degraders.

**Figure 12:**
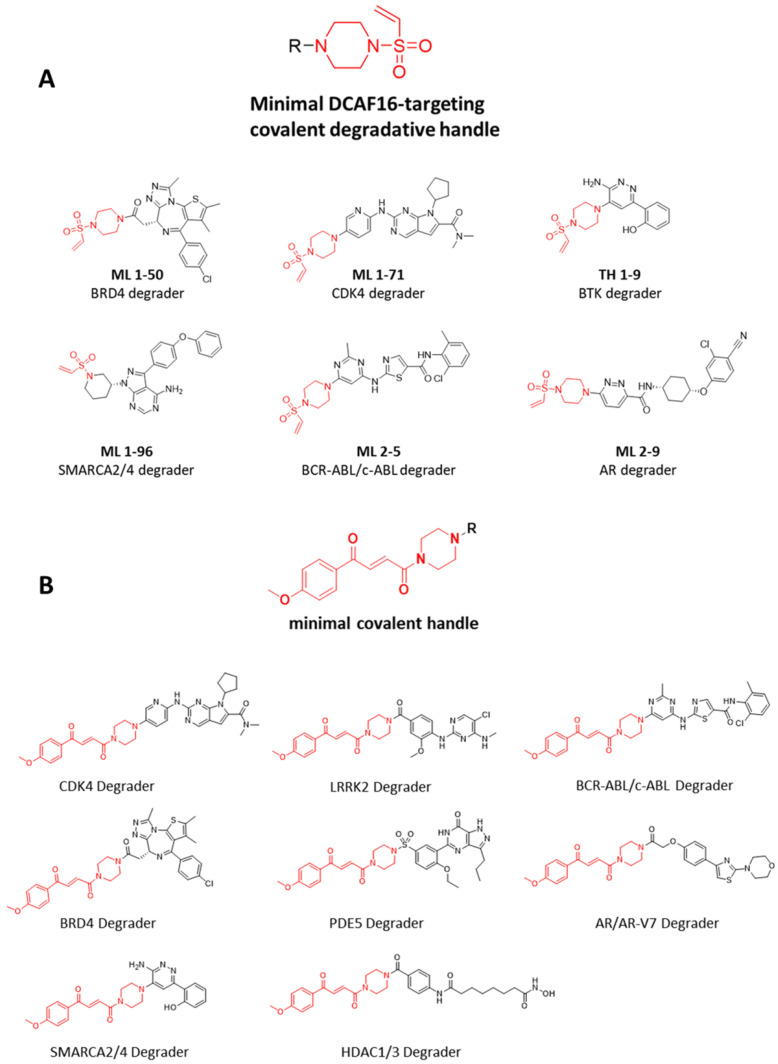
The work of Nomura’s group: (**A**) vinylsulfonyl piperazine covalent handle and its applications. (**B**) minimal covalent handle and its applications.

In mid-March 2023, Professor Nomura’s group at UC Berkeley reported their latest research on molecular glue degraders, identifying a transplantable covalent warhead—vinyl sulfonyl piperazine—that can be installed onto various target ligands to convert non-degrading binders into effective covalent degraders (Figure 12A) [141]. In the same journal, the group demonstrated a similar strategy, applying covalent warheads to ligands targeting a wide range of proteins including CDK4, LRRK2, BCR-ABL/c-ABL, BRD4, PDE5, BTK, SMARCA2/4, HDAC1/3, and AR/AR-V7, successfully enabling targeted protein degradation. (Figure 12B) [142]

**Figure 13:**
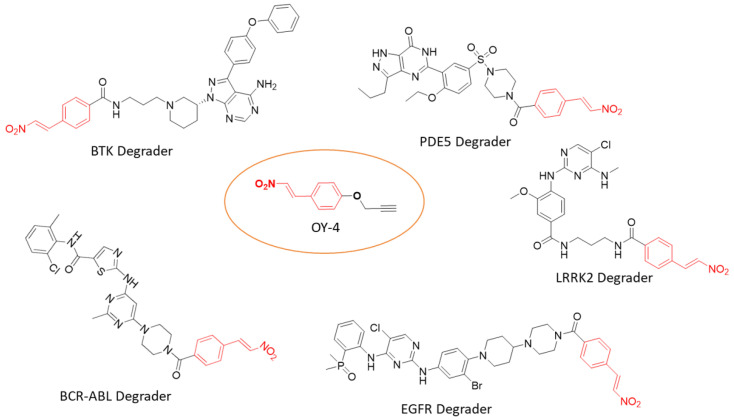
Work from the Ding and Li groups.

In June 2025, a collaborative team led by Ding and Li reported the use of β-nitrostyrene as a reversible cysteine-targeting electrophilic warhead. Using phenotypic screening and chemoproteomic platforms, the team identified novel druggable sites and developed covalent probes that regulate proliferation in acute myeloid leukemia cell lines. Incorporating this warhead into the BRD4 inhibitor (+)-JQ1 enabled recruitment of the E3 ligase TRIM28 and promoted targeted protein degradation. Furthermore, applying the same strategy to ligands for other targets—such as EFGR^L858R/T790M/C797S^, PDE5, BTK, LRRK2, BCR-ABL/c-ABL, effectively induced degradation of the proteins of interest without provoking hook effects. (Figure 13) [143]

The most prominent advantage of this strategy lies in providing a relatively clear pathway to repurpose a vast array of existing and well-characterized protein inhibitors into degraders with novel mechanisms of action. However, significant challenges remain, including the rational selection of attachment sites, optimization of linker length and chemical properties, and accurate prediction of interactions with specific E3 ligases. Additionally, while the use of covalent warheads can enhance potency and selectivity, it may also introduce risks such as immunogenicity or off-target covalent binding to unintended proteins. Furthermore, the effectiveness of this approach is highly dependent on the availability of suitable E3 ligases. Currently, most research is focused on a limited set of E3 ligases, such as CRBN and DCAF16. Future efforts should therefore prioritize the discovery of ligands for a broader range of E3 ligases to expand the applicability and versatility of this strategy.

## 7. Challenges and Prospect

Over the past two decades, research on molecular glue degraders (MGDs) has indeed undergone a profound transformation—from serendipitous discovery to rational design—with its evolution clearly mirroring the rapid advancement of the targeted protein degradation field from proof-of-concept to clinical application [144].

In the early 21st century, the study of MGDs largely originated from a renewed understanding of the mechanisms of action of certain existing drugs. Research during this period focused on elucidating how these molecules “reprogram” E3 ubiquitin ligases, enabling them to recognize and ubiquitinate novel substrate proteins, leading to their subsequent degradation via the proteasomal pathway. The most representative example is the elucidation of the mechanisms of thalidomide and its derivatives—IMiDs such as lenalidomide and pomalidomide [145,146].

As the understanding of E3 ligase mechanisms, particularly those involving CRBN, deepened, research entered a phase of accelerated development. Through approaches such as phenotypic screening and chemical proteomics, molecular glues capable of degrading diverse targets including GSPT1 [147,148], CK1α [149], and BRD9 [150] were successively identified. New methodological frameworks were also developed to systematically discover and characterize MGDs. For instance, integrating genetic code expansion technology with mass spectrometry-based proteomics enables global cross-linking analysis of the E3 ligase interactome induced by molecular glues in living cells, facilitating efficient identification of novel substrates [151]. Artificial intelligence, molecular docking, and molecular dynamics simulations have also been employed to predict and elucidate the mechanisms of natural products—such as bufalin—that function as molecular glues [138].

In recent years, the field has shifted most notably from serendipitous discovery toward rational design, with an accelerated push toward clinical translation. Researchers have begun to delineate design principles aimed at converting known protein ligands into molecular glue degraders. One strategy involves appending specific “gluing” modules—such as hydrophobic aromatic rings, double bonds, or covalent warheads—to inhibitors, enabling additional recruitment of E3 ligases (e.g., DCAF16) and thereby transforming inhibitors into degraders [142,152]. Another approach entails modifying CRBN-binding scaffolds (e.g., heteroaryl glutarimides and dihydrouracils) through scaffold hopping and structural optimization to enhance stability and degradation selectivity. The therapeutic application of MGDs has expanded beyond hematological malignancies to solid tumors and is being explored in combination with immunotherapies. For example, PVTX-405, a molecular glue degrader of IKZF2, can relieve the immunosuppressive effects of regulatory T cells in the tumor microenvironment; when combined with anti–PD-1 or anti–LAG-3 therapy, it significantly enhances antitumor efficacy [153]. Likewise, strategies that degrade SPOP to stabilize STING protein can also synergize with immune checkpoint blockade or CAR-T therapy [154].

### 7.1. Challenges

However, the development of MGDs still faces several challenges that hinder their broader application.

#### 7.1.1. The Diversity of E3 Ligases Employed by Molecular Glues Remains Limited

The diversity of E3 ligases exploitable by molecular glues remains limited, with the majority of research focused predominantly on CRBN. Despite its dominance, researchers in the field have recognized the importance of expanding the E3 ligase toolbox and have begun exploring alternative possibilities. However, the discovery of molecular glues that engage E3 ligases beyond CRBN still faces considerable challenges. This is largely because molecular glue function depends on inducing novel, high-affinity interaction interfaces between proteins—principles that remain poorly understood for most E3 ligases. Unlike conventional inhibitors that occupy well-defined active sites, molecular glues typically do not require deep binding pockets on the target protein, rendering their rational design particularly difficult [1,155].

Currently, researchers are employing multiple strategies to advance molecular glue discovery, including repurposing known E3 ligase ligands, screening for novel binders against proteins of interest, and applying functional genomics and quantitative proteomics to elucidate molecular glue mechanisms. For instance, the combination of genetic code expansion technology with mass spectrometry-based proteomics enables global profiling of molecular glue-induced E3 ligase interactomes in living cells, offering a promising route to identify new E3–glue–substrate combinations [1,151].

#### 7.1.2. MGDs Research Remains Heavily Skewed Towards Oncology

MGDs, as a revolutionary targeted protein degradation strategy, have seen their research fervor primarily driven by the demands of cancer therapy. This focus is rooted in the field’s historical origins, scientific maturity, and clinical successes, as many cancer cells critically depend on specific “undruggable” proteins for survival and proliferation. For instance, GSPT1, a small GTPase involved in translation termination, has been shown to promote cancer progression, making it a highly attractive therapeutic target for MGDs. The success of molecular glues engaging E3 ubiquitin ligases such as CRBN in hematological malignancies—exemplified by lenalidomide and its derivatives—has significantly galvanized investment in this area within oncology, creating a virtuous cycle from fundamental research to clinical validation [156,157].

Future development will likely need to focus on the following aspects: **Deepening mechanistic exploration**: Drawing on the refined mechanistic insights gained from oncology targets such as GSPT1 and PP2A, systematic efforts should be made to identify and validate analogous “node” proteins in non-oncological diseases that are amenable to molecular glue intervention. **Migration of technological platforms**: Established discovery tools from oncology research—including structure-based rational design, chemical proteomics, and computational modeling—should be adapted for the screening and optimization of non-oncological targets. **Cross-disciplinary collaboration**: Strengthening collaborative networks between oncology researchers and experts in neuroscience, immunology, and infectious diseases will be essential. Future breakthroughs will depend on a deeper understanding of non-oncological disease biology, the establishment of interdisciplinary collaborative networks, and the creative application of technological platforms refined in oncology to new therapeutic contexts. Through deliberate strategic planning, the research landscape of molecular glues—currently heavily skewed toward oncology—can gradually evolve toward a more balanced and comprehensive trajectory, ultimately benefiting a broader range of patient populations.

#### 7.1.3. Molecular Glue Degraders Can Induce Toxicity Due to Off-Target Effects

Many MGDs may interact with off-target proteins, leading to potential toxicity. CRBN, as a key substrate recognition subunit of the E3 ubiquitin ligase complex, indeed presents significant risks and challenges in exploring its chemical space. These risks arise not only from its complex biological functions but also directly lead to various obstacles in the clinical development of immunomodulatory drugs (IMiDs), represented by thalidomide and its derivatives, resulting in slow progress.

Teratogenicity, defined as the risk of inducing congenital malformations during embryonic development by drugs, is a core safety challenge faced by small molecular drugs targeting the Cereblon E3 ubiquitin ligase, including classical immunomodulatory drugs and their derivative protein degradation-targeting chimeras. This risk is particularly salient in non-oncological indications, where long-term medication is required, and the patient population may include individuals of childbearing age [158,159].

The degradation profile of “new substrates” induced by drug binding to CRBN is crucial in determining its biological effects, including efficacy and toxicity. When thalidomide and its analogs bind to CRBN, they alter the substrate specificity of this E3 ligase, leading to the ubiquitination and degradation of a range of proteins not originally targeted. Among the numerous newly degraded substrates, the degradation of the transcription factor SALL4 has been identified as a key event directly linked to teratogenic phenotypes in humans. Research indicates that thalidomide and its analogs induce SALL4 degradation via CRBN, which is directly related to teratogenicity in susceptible non-clinical species [160]. Importantly, SALL4 degradation is likely not the sole mechanism by which thalidomide induces teratogenicity. CRBN is widely expressed across various human tissues and organs, and its normal physiological functions, particularly its endogenous substrate spectrum, have yet to be fully elucidated. This implies that, beyond known new substrates (such as SALL4, Ikaros, CK1α, etc.), drugs may interfere with other unknown functions of CRBN, posing potential risks. Therefore, a comprehensive assessment of teratogenic risk requires attention beyond individual proteins [161].

**Figure 14:**
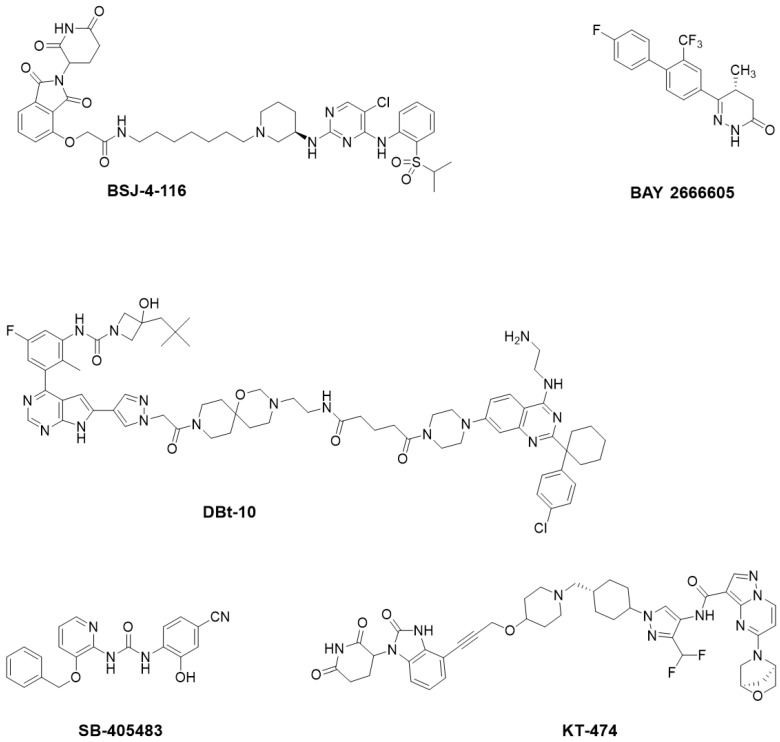
The chemical structure of **BSJ-4-116**, **BAY 2666605**, **DBt-10, SB-405483 and KT-474**.

Given the mechanism-driven characteristics of teratogenic risk, it has become possible to mitigate these risks through rational drug design and rigorous preclinical screening. Current strategies primarily focus on the following aspects:

Design of Novel Bifunctional Molecules: The core design goal here is to maintain efficient degradation of therapeutic targets while avoiding the degradation of new substrates associated with teratogenicity, such as SALL4. Recent studies, including negative results from some protein degradation-targeting chimeras in embryonic-fetal assays, strongly demonstrate that structural optimization can lead to molecules with sufficient specificity to avoid teratogenic risk. For instance, the newly designed bifunctional degraders like KT-474 (Figure 14) aim to circumvent the degradation of new substrates associated with immunomodulatory drugs. In vitro proteomic analyses confirm that KT-474 selectively degrades its target protein IRAK4 without affecting SALL4 or other detected CRBN new substrate levels [162,163].

Development of Novel E3 Ligands: The limitations of CRBN ligands based on the classic immunomodulatory drug framework, including teratogenic risks from degrading SALL4, highlight the importance of developing E3 ligands with new chemical structures. Research has successfully designed non-cyclopentyl amide-based CRBN ligands for constructing protein degradation-targeting chimeras aimed at BRD4 and ALK [164]. These new ligands not only reduce degradation of immunomodulatory drug-related new substrates but also induce entirely new degradation profiles of CRBN substrates, potentially separating teratogenic risk from therapeutic efficacy.

Compound Design Targeting Allosteric Sites on CRBN: Recent breakthrough studies have identified a hidden, evolutionarily conserved allosteric binding site on CRBN for the first time [132]. They discovered a small molecule, SB-405483 (Figure 14), that binds to this site and tested its effects on the degradation of six different substrates in combination with hundreds of orthosteric ligands, including molecular glues and PROTACs. The results indicated that SB-405483 generally enhanced the degradation of substrates like CK1α, Wee1, and GSPT1; however, it often inhibited the degradation of zinc finger protein substrates like IKZF1/3, IKZF2, and SALL4. This suggests that allosteric modulation is substrate-selective, and future research could achieve “precise degradation” by pairing different orthosteric and allosteric ligands, thus avoiding harm to innocent proteins.

In addition to teratogenicity, the development of CRBN chemical space faces multiple risks that collectively contribute to the slow and complex clinical development of drugs such as IMiDs. The first of these is off-target effects and unintended degradation of new substrates. CRBN is widely expressed in various tissues and organs throughout the human body. While IMiDs degrade expected targets, such as IKZF1/IKZF3 in multiple myeloma, they inevitably also degrade a range of other “new substrates.” [164,165] For instance, in megakaryocytes, IMiDs disrupt the normal degradation of thrombospondin-1 (THBS-1) by CRBN, leading to abnormal accumulation of THBS-1 [166], which is considered an important pathogenic mechanism for the thromboembolic risks associated with IMiD therapy. In certain diseases, the degradation of different new substrates may contribute separately to efficacy or toxicity. For example, in primary effusion lymphoma, the degradation of CK1α is a key effect related to the toxicity of IMiDs, while the degradation of IKZF1/IKZF3 is not essential. This complicates the design of drugs with a wide therapeutic window [167].

The second risk is the inherent limitations of the chemical scaffold of CRBN ligands. The IMiD scaffold exhibits hydrolytic instability, which may lead to suboptimal drug metabolism and pharmacokinetic properties. The reliance on the traditional IMiD structure restricts the expansion of the chemical space and target range for CRBN-recruiting PROTACs [164].

Such issues may be addressed through improved affinity-based screening technologies and the development of novel MGD modalities, such as Molecular Glue Antibody Conjugates (MACs) [168] and ProDegraders [169]. WuXi AppTec has established a DEL-based MGD discovery platform and an affinity selection mass spectrometry (ASMS) platform, aiming to identify compounds with higher specificity through advanced screening and structure-guided SAR studies. As the field evolves, researchers are leveraging antibody-drug conjugate (ADC) strategies [170,171]—akin to those used with traditional cytotoxic ADCs—to mitigate toxicity resulting from insufficient specificity. MACs combine the targeted delivery of antibodies with the protein degradation capability of MGDs. A linker connects the molecular glue degrader to the antibody, enhancing stability in systemic circulation while ensuring efficient release of the degrader at the tumor site. This innovative approach may improve therapeutic efficacy and reduce systemic toxicity, representing a promising direction for patients. The ProDegrader strategy, which resembles a prodrug approach, employs intramolecular cyclization to form a glutarimide moiety that induces degradation. This method is broadly applicable to CRBN-recruiting degraders, allowing direct conjugation of the uncyclized ProDegrader. Upon cyclization, the active degrader is released in its unmodified form, preserving potency. This strategy has also been applied in degrader-antibody conjugates (DACs) [172,173,174].

#### 7.1.4. The Potential Mechanisms of Resistance to Molecular Glue Degraders

While MGDs hold promise for overcoming resistance to traditional inhibitors, they may also trigger resistance themselves. The mechanisms of such resistance can be classified into two main types: resistance mediated by target protein mutation, and resistance due to downregulation or mutation of the E3 ligase.

Resistance caused by target protein mutation represents the most straightforward mechanism. Using techniques such as CRISPR screening, mutations conferring resistance have been identified in neo-substrates of molecular glues [174]. These mutations fall primarily into two categories: the first directly interferes with ternary complex formation, where mutations at the interface between the target protein and either the molecular glue or the E3 ligase destabilize the complex and prevent degradation [175]. The second involves distal mutations that exert subtle yet sufficient effects: some mutations located outside the binding interface may allosterically reduce degradation efficiency. Research indicates that even a slight decline in degradation efficiency can enable cell survival and confer a resistant phenotype. For example, in the case of the CDK12 degrader **BSJ-4-116** (Figure 14), two point mutations in CDK12 were found to confer resistance to the compound [176].

Tumor cells may also evade degradation by downregulating E3 ligase expression. Studies have shown that CRBN, a commonly used E3 ligase in targeted protein degradation, can be downregulated in the context of resistance to immunomodulatory drugs. This has spurred the development of degraders based on alternative E3 ligases, such as DCAF1, to overcome such resistance. This mechanism is E3 ligase-specific; studies confirm that cells with acquired resistance to a CRBN-based BTK PROTAC can still be effectively treated with a DCAF1-based BTK degrader **DBt-10** (Figure 14). This suggests that resistance may often be E3 ligase-specific, underscoring the importance of maintaining a diverse E3 ligase toolkit as a key counter-strategy [177].

#### 7.1.5. Designing MGDs to Effectively Penetrate the BBB for CNS Targets Remains a Significant Challenge

The blood–brain barrier (BBB) is a crucial physiological structure that protects the brain, yet it also severely restricts the entry of most large molecules and many small-molecule drugs into the central nervous system (CNS). For emerging targeted protein degradation strategies such as molecular glues, the ability to cross this barrier is pivotal to their successful application in neurodegenerative diseases or brain tumors [178].

Encouragingly, recent preclinical studies on brain tumors have provided direct evidence that rationally designed molecular glue compounds can achieve BBB penetration. For instance, in glioblastoma research, a molecular glue named **BAY-2666605** [179] (Figure 14) not only demonstrated antitumor activity in preclinical models but was also clearly shown to cross the BBB. In an orthotopic xenograft model using GB1 cells, this compound induced complete tumor regression, directly proving its ability to reach therapeutically effective concentrations within the CNS. This success offers important proof of principle and builds confidence for applying molecular glues to other CNS disorders, such as Alzheimer’s disease and Parkinson’s disease [180].

Despite these individual successes in BBB penetration, how to systematically design molecular glues with favorable BBB permeability remains a key challenge in the field. Current potential strategies to address this issue may include: Optimizing physicochemical properties: Drawing on experience from traditional small-molecule neurotherapeutic design, parameters such as molecular weight, lipophilicity, and the number of hydrogen-bond donors/acceptors can be modulated to enhance passive diffusion. Utilizing endogenous transport systems: Molecular glues could be designed to mimic the structure of nutrients or specific substrates, thereby hijacking active transport systems present at the BBB to facilitate brain entry. Developing novel CNS-directed E3 ligase ligands: This represents a unique opportunity in molecular glue design. Traditional molecular glues rely on E3 ligases such as cereblon, whose distribution and function in the CNS may not be optimal. Identifying and developing ligands for novel E3 ligases that are highly expressed and functionally specific in the brain could enable the next generation of molecular glues—ones that not only degrade target proteins efficiently but also possess inherently favorable CNS distribution.

### 7.2. Prospect

Based on the current research landscape and challenges associated with molecular glue degraders, we propose that future studies should prioritize the following areas:

#### 7.2.1. Translation and Extension of TPD Technology Platforms

**Figure 15:**
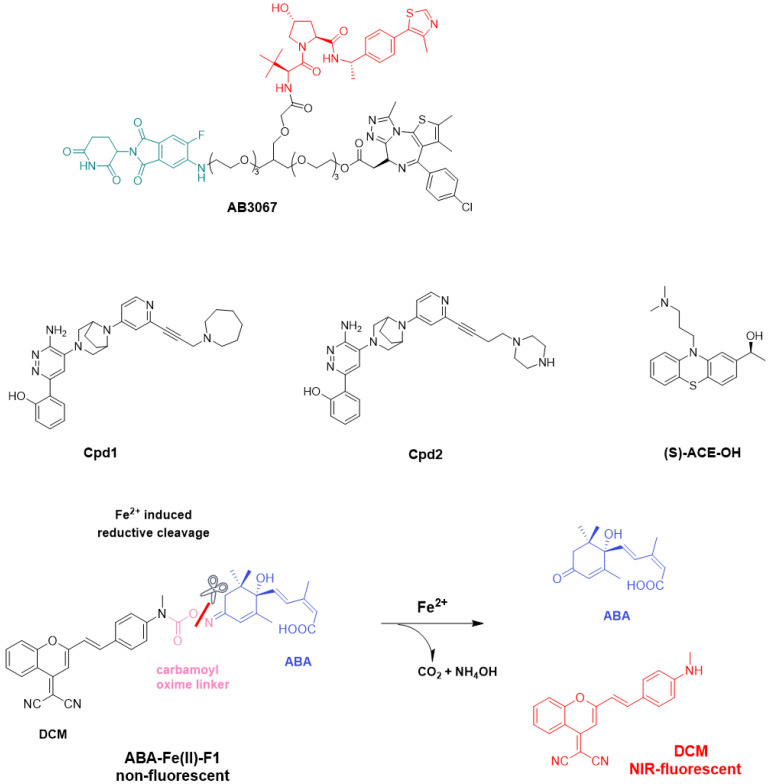
The examples in the Section 7.2 involve the chemical structure of compounds.

The translation and extension of TPD technology platforms refers to the application of existing TPD molecule discovery and design methods to the development of molecular glue degraders. A notable example is the evolution from multivalent PROTAC design to molecular glue degrader development. For instance, the team led by Ciulli developed **AB3067** (Figure 15), a heterotrivalent PROTAC composed of CRBN, VHL, and BET-targeting ligands, which emerged as one of the most potent and rapid degraders of BET proteins while minimizing cross-degradation of E3 ligases [181].

Although this study preliminarily validated the feasibility of dual-ligase recruitment through structurally complex “trivalent PROTACs,” it presented a significant challenge for simpler, more drug-like “monovalent molecular glues.” Encouragingly, the Winter & Ciulli team subsequently discovered and characterized **Cpd1** (Figure 15), a monovalent molecular glue degrader capable of recruiting two distinct E3 ligases, DCAF16 and FBXO22, to degrade SMARCA2/4. The study elucidated its unique covalent mechanism of action, which involves forming a bond with C173 within a flexible loop of DCAF16. Importantly, the research demonstrated that this dual-ligase dependency is “tunable”: not only can dependency be switched between DCAF16 and FBXO22 through subtle chemical modifications of the degrader **Cpd2** (Figure 15), but the response to a specific degrader can also be enhanced via site-directed mutation of the E3 ligase (e.g., DCAF16 ^L59W^). These findings provide new strategic and theoretical foundations for the rational design of next-generation targeted protein degraders with the potential to overcome resistance and exhibit greater therapeutic efficacy [182].

However, it should be noted that the discovery process for such molecules was not detailed in the study, suggesting that systematic discovery methodologies for this class of degraders may still be in early stages and require further refinement through additional research.

#### 7.2.2. Development of Novel Delivery Systems to Enhance Tissue Targeting

The development of novel delivery systems to improve the tissue targeting of molecular glue degraders represents a highly promising research direction in the field of TPD. MGDs commonly face challenges in clinical translation, including poor water solubility, potential systemic toxicity, and inefficient delivery. Therefore, constructing intelligent and precise delivery systems is crucial for unlocking their therapeutic potential and advancing their clinical application [183].

Enhancing tissue targeting through innovative delivery platforms is a multidisciplinary frontier. Localized sustained-release hydrogels offer a direct and safe approach for solid tumor therapy, while pre-targeting strategies based on high-affinity molecular pairs (such as Barnase–Barstar) and biomimetic cell-based technologies open new avenues for precise localization after systemic administration. Additionally, the guidance of computational models enables the rational design of strategies to improve cytosolic delivery [184,185].

Future research is expected to move toward more intelligent and sophisticated “all-in-one” delivery systems, potentially integrating multiple strategies—for example, by conjugating active targeting ligands to hydrogel or exosome-based carriers—and responding to tumor microenvironment-specific stimuli (such as pH, enzymes, or redox status) for on-demand drug release. Through continued optimization of these delivery technologies, it is anticipated that current delivery bottlenecks for molecular glue degraders can be overcome, ultimately facilitating their successful clinical translation in the treatment of cancer and other diseases.

#### 7.2.3. Exploration of E3 Ligase-Independent Degradation Strategies

Traditional molecular glue degraders (MGDs) primarily function by hijacking E3 ubiquitin ligases to induce proximity between a target protein and the E3, leading to ubiquitination and subsequent proteasomal degradation. However, this paradigm has limitations, as certain proteins may develop resistance to E3-mediated degradation [186,187].

E3 ligase-independent degradation does not represent a single pathway but encompasses a range of distinct molecular mechanisms, from specialized enzymatic functions to entirely novel degradation routes. In recent years, a series of studies has revealed new protein degradation mechanisms that operate independently of canonical E3 ligase activity. These “E3 ligase-independent” strategies offer fresh perspectives and tools to overcome existing challenges and expand therapeutic approaches [186]. For example: under conditions of intracellular GTP depletion, the nucleolar protein NS is degraded by the proteasome via a mechanism that does not require ubiquitin or the E3 ligase MDM2. Even inactivation of the ubiquitin-activating enzyme E1 fails to prevent NS degradation, clearly confirming an independent proteasomal degradation pathway that bypasses the classical ubiquitination system [188]. Certain plant pathogens (e.g., phytoplasmas) have evolved E3-independent proteasomal degradation mechanisms to dismantle host targets. This provides inspiration for developing novel targeted degraders that do not rely on host E3 machinery [186].

Beyond these, aberrant polymer degradation represents another E3 ligase-independent mechanism. Research led by Han Ting’s team found that the metabolite (S)-ACE-OH (Figure 15), derived from levomepromazine, exhibits molecular glue activity by inducing an interaction between the E3 ubiquitin ligase TRIM21 and the nucleoporin NUP98, leading to the degradation of the nucleoporin. Based on this, levomepromazine-derived TrimTAC molecules can selectively degrade pathogenic protein aggregates without affecting their monomeric forms. Given that abnormal protein aggregation is implicated in various diseases—including autoimmune disorders, neurodegenerative conditions, and cancer—this research highlights the broad therapeutic potential of polymer-targeted degradation technology [189].

#### 7.2.4. Expansion of Molecular Glue Applications in Non-Oncological Diseases

As the technology matures, molecular glues are poised to find broader application in areas such as neurodegenerative diseases, autoimmune disorders, and metabolic diseases, offering novel therapeutic options for these conditions.

MGDs technology presents new therapeutic possibilities for neurodegenerative diseases, particularly in conditions like Alzheimer’s disease and Parkinson’s disease. Research in 2025 has shown that molecular glues can target disease-associated proteins, such as Tau and α-synuclein, promoting the degradation of these pathogenic proteins and thereby slowing disease progression [190,191]. For instance, the strategic collaboration between Eisai and Seed Therapeutics, a subsidiary of BeyondSpring, aims to discover, develop, and commercialize novel molecular glue degraders for multiple neurodegenerative disease targets. Under the agreement, Seed Therapeutics plans to advance in vivo efficacy studies for a Tau-targeted degrader project (for Alzheimer’s disease) in 2025, followed by an IND application in 2026. Additionally, the strategic collaboration between Roche and Monte Rosa Therapeutics also includes the development of molecular glue degraders for neurological disease targets. These partnerships underscore the growing recognition of the potential of molecular glue technology in treating neurodegenerative disorders, offering hope for new therapeutic approaches in areas where effective treatments remain limited.

In the field of autoimmune diseases—conditions such as rheumatoid arthritis, systemic lupus erythematosus, and multiple sclerosis, which result from the immune system mistakenly attacking the body’s own tissues—traditional treatments often rely on non-specific immunosuppressants that may cause significant side effects. Molecular glue technology offers a new strategy, particularly by targeting key proteins that regulate immune cell function. In 2025, preclinical studies of MRT-6160, a VAV1-targeted molecular glue degrader developed by Monte Rosa Therapeutics, demonstrated promising activity, suggesting its potential as a treatment for various immune-mediated diseases, including multiple sclerosis, rheumatoid arthritis, inflammatory bowel disease, and dermatological conditions [47].

For metabolic diseases such as diabetes and obesity, which represent major global health challenges with limited treatment options, molecular glue technology also offers new avenues. In March 2025, researchers at Mount Sinai identified a molecular glue that helps protect insulin-producing cells from diabetes-related damage. By modulating specific protein–protein interactions, these molecules reduce inflammatory responses, preserve pancreatic β-cell function, and thereby improve insulin secretion and glycemic control. Efforts are now underway to optimize these compounds and evaluate their clinical translation potential. Future research will focus on refining therapeutic molecular glues and testing them in preclinical models of diabetes. This work opens new paths for developing innovative drugs to treat diabetes, potentially improving patient outcomes and quality of life [192].

#### 7.2.5. Integration of Molecular Glues with Imaging Technologies

Integrating molecular glues with imaging technologies to develop theranostic molecular glues represents a highly promising and significant innovation in current biomedical research. The core of such theranostic molecular glues lies in their modular design, which typically integrates three key functions into a single molecule or nanosystem. These include: A molecular-glue functional module responsible for inducing or modulating protein–protein interactions, such as inducing target protein degradation or controlling protein localization; An imaging/reporter module, usually a fluorophore, for real-time and in situ reporting of the activation status or therapeutic effect of the molecular glue, enabling visual monitoring; and A linkage-and-response module that connects the above two parts via specific chemical bonds or responsive linkers, capable of releasing the active molecular glue upon triggering by specific biological signals (e.g., metal ions, enzymes), thereby achieving spatiotemporally controlled activation. For example, one study reported an abscisic acid (ABA)-based theranostic molecular glue, ABA-Fe(II)-F1 (Figure 15). It links ABA with a near-infrared fluorophore DCM through a unique carbamoyl oxime linker. This molecular glue can specifically sense intracellular Fe^2+^, resulting in turn-on near-infrared fluorescence for imaging, while simultaneously releasing active ABA molecules to regulate downstream gene expression and protein trafficking [193].

Such a design enables researchers not only to intervene in cellular processes but also to visualize where, when, and to what extent the intervention occurs, allowing dynamic tracking and precise manipulation of biological processes. In the future, with the integration of more biocompatible responsive linkers and advanced imaging modalities, theranostic molecular glues are expected to play an increasingly critical role in the precise diagnosis and treatment of major diseases such as cancer and neurological disorders, while also providing unprecedented dynamic analytical tools for fundamental biological research.

## 8. Conclusions

MGDs are poised to become a pivotal pillar in drug discovery, offering novel strategies and approaches for treating a wide range of diseases. With continuous technological advancement and refinement, molecular glues will enable the targeting of an expanding array of “undruggable” proteins, achieving more precise and effective therapeutic interventions and bringing new hope to patients worldwide.

In summary, the emergence of molecular glue technology represents a major revolution in the field of drug development. It not only significantly expands the scope of targetable proteins but also provides innovative concepts and methodologies for the treatment of diverse diseases. As research deepens and technology progresses, molecular glue-based strategies are set to play an increasingly vital role in future medical practice.

## Data Availability

No new data were created or analyzed in this study. Data sharing is not applicable to this article.
